# The Effects of Anti-Vascular Endothelial Growth Factor Drugs on Retinal Pigment Epithelial Cell Culture

**DOI:** 10.4274/tjo.20270

**Published:** 2018-09-04

**Authors:** Mustafa Şahiner, Dilek Bahar, Ayşe Öner, Zeynep Burçin Gönen, Metin Ünlü, Duygu Gülmez Sevim, Çağatay Karaca, Galip Ertuğrul Mirza

**Affiliations:** 1 Erciyes University Faculty of Medicine, Department of Ophthalmology, Kayseri, Turkey; 2 Erciyes University Betül-Ziya Eren Genome and Stem Cell Center, Kayseri, Turkey; 3 Erciyes University Faculty of Dentistry, Department of Oral and Maxillofacial Surgery, Faculty of Dentistry, Kayseri, Turkey

**Keywords:** Anti-VEGF, cell culture, senescence, retinal pigment epithelial cell

## Abstract

**Objectives:**

To assess the effects of anti-vascular endothelial growth factor (VEGF) drugs on retinal pigment epithelium cell culture.

**Materials and Methods:**

Aflibercept (0.5 mg/mL), bevacizumab (0.3125 mg/mL), and ranibizumab (0.125 mg/mL) were applied to retinal pigment epithelium cell cultures isolated from the enucleated eyes of New Zealand white rabbits. Viability, apoptosis, proliferation, and senescence of the cells were evaluated in control and drug-treated cultures at the end of 72 hours.

**Results:**

Cells treated with aflibercept showed increased viability and decreased apoptosis compared to the control culture and both the bevacizumab- and ranibizumab-treated groups (p<0.05). Statistically increased apoptosis and decreased viability were found in the bevacizumab and ranibizumab-treated groups compared with the control group (p<0.05). There were no statistically significant differences in cell proliferation and senescence between the groups (p>0.05).

**Conclusion:**

Anti-VEGF drugs did not affect senescence or proliferation of retinal pigment epithelium cells. Aflibercept was found to decrease apoptosis and increase cell viability, while ranibizumab and bevacizumab increased apoptosis and reduced cell viability in retinal pigment epithelium culture.

## Introduction

It is well known that vascular endothelial growth factor A (VEGF-A) is a main mediator of angiogenesis and increased vascular permeability in retinal vascular disorders.^1,2,3,4^ The inhibition of vascular endothelial growth factor (VEGF) has been a key point in experimental and clinical studies under research. The effectiveness of intravitreal administration of various anti-VEGF agents is well established in the treatment of macular edema of different origins.^5^ The mechanism of action of these drugs when delivered intravitreally is complex and involves the blocking of various types of VEGFs, decreased permeability of newly formed blood vessel walls, and reduced swelling of the retinal layers. In recent years, several reports have demonstrated the impact of anti-VEGF drugs upon different cell cultures in vitro.^6,7,8,9,10,11^ Our goal was to investigate the effects of anti-VEGF drugs on viability, apoptosis, proliferation, and senescence in retinal pigment epithelium (RPE) cell culture, which can serve as an in vitro model.

In this study, we compared the proliferative and cytotoxic effects of aﬂibercept (0.5 mg/mL), bevacizumab (0.3125 mg/mL), and ranibizumab (0.125 mg/mL) on RPE cell cultures by evaluating viability, apoptosis, proliferation, and senescence in control and drug-treated cells after 72 hours.

## Materials and Methods

### Animals

Eyes were obtained from 4 New Zealand white rabbits that weighed between 1.5 and 2.2 kg. Animal care and surgical procedures were attempted in scrupulous agreement with the approval of the Ethical Committee of Erciyes University (TTU-2015-5996). The rabbits were killed by injecting a lethal dose of ketamine/xylazine. The globes were enucleated and placed in Ca^2+^ and Mg^2+^-free phosphate buffered saline augmented with penicillin/streptomycin (GIBCO, 15140-0122).

### Isolation and Culture of Rabbit Retinal Pigment Epithelium

Rabbit RPE cells were isolated and maintained as described by Chang et al.^12^ After incubating the globes with 2% dispase for 15 minutes, an incision was made 3 mm from the limbus and continued circumferentially. After removal of the cornea and lens, 4 radial incisions were made in the posterior segment, and this part was incubated in Dulbecco’s modified Eagle’s medium/Ham’s F12 (DMEM/ F12) medium augmented with 10% fetal bovine serum for 2 hours. Finally, the RPE cells were separated from the neural retina and choroid as a sheet with micropipettes and observed under a stereo microscope (Olympus BX51, Japan). Passage 3 cells were used for the study and drugs were applied to the cultures 24 hours after fresh cell plating.

Ranibizumab (Lucentis, Novartis, Switzerland), a fragment of a human monoclonal antibody against VEGF-A selectively binds all isoforms of VEGF-A (VEGF110, VEGF121, and VEGF165), was applied at a concentration of 0.125 mg/mL. Bevacizumab (Avastin, Genetech/Roche, USA), a monoclonal antibody against VEGF which is used off-label to treat various eye diseases, was added to the cultures at a concentration of 0.3125 mg/mL. Aflibercept (Eylea, Bayer Health Care, Germany), a fusion protein that binds to circulating VEGF (subtypes VEGF-A and VEGF-B) and placental growth factor (PGF), was used at a concentration of 0.5 mg/mL.

### Immunocytochemistry Staining

For immunofluorescence staining, the RPE cells were fixed with 0.4% paraformaldehyde in PBS and permeabilized with 0.4% Triton X-100. Bovine serum albumin (10 mg/mL) was used as a stabilizing agent for proteins such as antibodies and enzymes. The cells were incubated overnight with primary antibody (zonula occludens protein 1 [Zo-1] invitrogen, 330100 and cytokeratin 18 chemicon, MAB3234). Labeled cells were detected with secondary antibodies for 1 hour, after each incubation, cells were washed with PBS 3-5 times, 5 minute each wash and assessed under a fluorescent microscope (Olympus BX51, Japan).

### MTT Proliferation Analysis

The 3-(4,5-dimethylthiazol-2-yl)-2,5-diphenyltetrazolium bromide (MTT) analysis is an established assay for evaluating metabolic activity of cells, and can be useful for the measurement of cell viability. MTT analysis was accomplished as previously reported for determining the metabolic activity of RPE cells.^13^ The medium was removed, the cells were washed with PBS, 1000 mL/well MTT solution was added, and the cells were incubated at 37 °C for 1 hour. The formazan crystals were dissolved after the administration of DMSO (1000 mL/well). Absorption was evaluated by a scanning spectrophotometer (Promega Glomax MultiDetection System Plate reader, USA) at 560 nm. The procedures were carried out 3 times. Treatment-naive RPE of the same passage served as the control.

### Senescence-associated β-galactosidase Activity

The percentage of RPE cells for positive senescence-associated (SA) β-galactosidase activity was detected as previously reported by Dimri et al.^14^ Concisely, treated RPE cells were washed twice with PBS and fixed with 2% formaldehyde and 0.2% glutaraldehyde in PBS (pH 6.0) at room temperature for 4 min. The cells were then washed twice with PBS and incubated for 8 hours at 37°C with freshly prepared SA β-galactosidase staining solution in darkness. Finally, SA β-galactosidase staining solution was eliminated, cells were washed with PBS, and the development of blueish color was visualized by light microscope.

### Detection of Apoptosis

The level of apoptosis was determined on the same cultures in which the cellular vital indices were analyzed. Apoptotic cells were identified by Muse™ Annexin V&Dead Cell Kit MCH100105 according to the manufacturer’s protocol. The cells were first washed in PBS and then detached for 10 minutes in trypsin and suspended again in PBS. Finally, the cells were centrifuged (1400 rpm, 5 minute) and resuspended in propidiumiodide (1 g/mL). The level of apoptosis was determined according to the number of apoptotic cells in pre-G1 phase using flow cytometry (Millipore Muse^®^ Cell Analyzer, Germany).

### Statistical Analysis

SPSS version 22.0 for Windows (Chicago, IL, USA) was used for statistical analysis. Descriptive data were presented as mean and standard deviation and percentages. Results were statistically analyzed by one-way ANOVA. P-values <0.05 were considered significant. All procedures were carried out 3 times.

## Results

### Evaluation of Retinal Pigment Epithelium Cell Morphology

**Figure 1:**
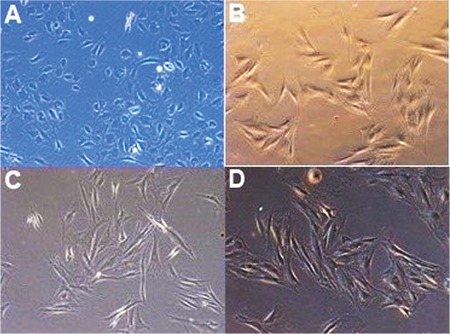
Comparison of phase-contrast microscopic appearance of 3 anti-vascular endothelial growth factor drugs and control culture showed no morphological changes of the retinal pigment epithelium (RPE) cell culture with any drug, and RPE cells maintain the hexagonal morphology at the end of 72 hours in the (A) control, (B) aflibercept (0.5 mg/mL), (C) bevacizumab (0.3125 mg/mL), and (D) ranibizumab (0.125 mg/mL) groups

In comparison of phase-contrast appearance of RPE culture cells 72 hours after supplementation with aflibercept (0.5 mg/mL), bevacizumab (0.3125 mg/mL), and ranibizumab (0.125 mg/mL), and control culture, phase contrast images showed no morphological changes in the RPE culture cells with any drug and RPE cells maintained their hexagonal morphology (Figure 1).

### Expression of Cytokeratin 18 and Zonula Occludens Protein 1

**Figure 2:**
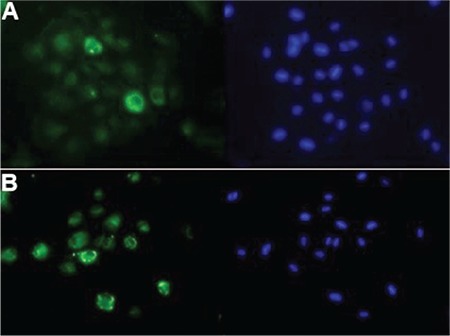
Immunocytochemistry staining of retinal pigment epithelium cell culture demonstrates expression of cytokeratin 18 (A) and tight junction protein zonula occludens protein 1 (B)

Immunocytochemistry images demonstrated expression of intermediate protein cytokeratin 18 (Figure 2A) and tight junction protein ZO-l (Figure 2B) in the RPE sheets.

### Effect of Anti-Vascular Endothelial Growth Factor Agents on Cell Viability

**Figure 3:**
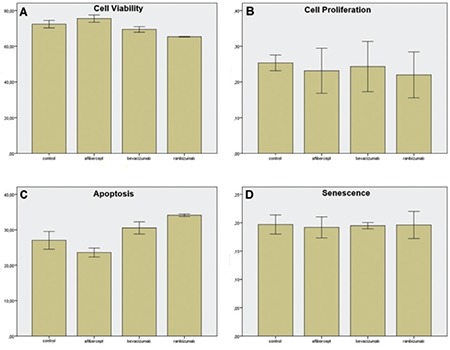
Bar graphs representing cell viability (A), cell proliferation (B), apoptosis (C), and senescence (D) in retinal pigment epithelium cell cultures 72 h after supplementation with aflibercept (0.5 mg/mL), bevacizumab (0.3125 mg/mL), and ranibizumab (0.125 mg/mL). A) Bevacizumab and ranibizumab decreased cell viability while aflibercept increased cell viability compared to the control group; B) None of the anti-vascular endothelial growth factor (VEGF) drugs showed a significant negative effect on cell proliferation; C) Bevacizumab and ranibizumab increased apoptosis while aflibercept significantly decreased apoptosis compared to the control group; D) None of the anti-VEGF drugs showed a significant effect on senescence

We attempted to assess if the 3 anti-VEGF drugs caused cytotoxic effects at the end of 72 hours. RPE cells were cultured until they reached 90% confluency and cytotoxicity was evaluated by MTT assay. There was significantly increased viability of the cells in the aflibercept-treated culture versus control culture (p=0.002) (Figure 3A). Statistically decreased viability was found in the bevacizumab and ranibizumab-treated groups versus control culture (p=0.003 and p=0.0001, respectively) (Figure 3A).

### Proliferation and Apoptosis of Retinal Pigment Epithelium Cells

Incubation for 72 hours with aﬂibercept (0.5 mg/mL), bevacizumab (0.3125 mg/mL), and ranibizumab (0.125 mg/mL) did not significantly affect cell proliferation when compared to control cell cultures (Figure 3B).

Apoptosis rates were significantly higher in the cultures treated with bevacizumab (0.3125 mg/mL) and ranibizumab (0.125 mg/mL) compared to the control group (p=0.001 and p=0.0001, respectively). There was a statistically significant decrease in the apoptosis of cells treated with aflibercept versus the control group (p=0.001) (Figure 3C).

### Senescence-associated β-galactosidase Activity

There was no statistically significant difference between the 3 anti-VEGF drugs at the end of 72 hours when evaluating their effects on cell senescence (p>0.05) (Figure 3D).

## Discussion

This is the first study in the literature to assess the senescence effects of anti-VEGF drugs and to identify that these agents do not seem to have a significant effect on the senescence of RPE cells in vitro. In addition, we have showed that RPE morphology and proliferation are also not affected by the anti-VEGF drugs most commonly used in retinal diseases. However, aflibercept increased viability and decreased apoptosis, while bevacizumab and ranibizumab had the opposite effect.

Intravitreal injection has become a widely used delivery route of various therapeutic agents for the treatment of vasoproliferative ocular diseases.^15^ Current anti-VEGF therapies delivered intravitreally include ranibizumab and aflibercept, as well as off-label bevacizumab. A main functional difference between aflibercept and other anti-VEGF agents is blockage of VEGF-B, PGF1, and PGF-2 in addition to VEGF-A isoforms. To best of our knowledge there are limited in vitro studies in the literature evaluating the safety and efficacy of ranibizumab, aflibercept, and bevacizumab. In the present study, aflibercept was found to decrease apoptosis and increase cell viability. In contrast, ranibizumab and bevacizumab were observed to increase apoptosis and reduce cell viability in RPE cultures. Retinal pigment epithelium-derived VEGF is a vital mediator for support of the choriocapillaris. Several clinical studies have shown a correlation between subfoveal choroidal thickness and RPE atrophy progression, which suggested that decreased blood supply might promote RPE atrophy during anti-VEGF treatment.^16,17^ We also noticed that most of the experimental studies on the adverse effects of the anti-VEGF drugs were carried out on RPE cell linings with intact choriocapillaris. Our study is performed on an isolated RPE cell line and may be able to show the direct toxic effects of anti-VEGF drugs on RPE. In a study performed on newborn rabbits, Cam et al.^18^ showed that at 3 weeks after injection, all anti-VEGF drugs caused low levels of serum anti-VEGF concentrations and induced apoptosis as determined with apoptotic index, which was described as the percentage of apoptotic TdT-mediated dUTP-digoxigenin nick end labeling (TUNEL) positive cells of tissues. Malik et al.^19^ also studied the safety profiles of various concentrations of anti-VEGF drugs on human RPE cells. They reported that while ranibizumab and aflibercept did not cause mitochondrial toxicity or cell death, bevacizumab and aflibercept revealed mild mitochondrial toxicity, though they also did not cause significant cell death at clinical doses. However, in our study all of the drugs caused a significant difference in cell viability. Malik et al.^19^ treated the culture media with anti-VEGF drugs at concentrations they considered to be the clinical dose, assuming the amount of intravitreal injected drug spreads equally throughout the 4 mL human vitreous and modifying the doses accordingly. No such modification was performed in our study, which is a limitation of our study and could explain our conflicting results.

In our study, anti-VEGF drugs had no effect on the senescence or proliferation of RPE cells at after 72 hours. Cellular senescence is a program activated by normal cells in reaction to various stress factors such as oxidative stress, DNA damage, oncogene activity, and inadequate culturing conditions.^20,21^ Conventionally, when cells enter senescence they exhibit substantial morphological changes. The cells spread out and flatten, which is usually followed by increasing SA β-galactosidase activity.^14^ To best of our knowledge there are no studies evaluating the effects of anti-VEGF drugs on senescence in the current literature. Our data showed that none of the anti-VEGF drugs affected senescence in RPE cells at the end of the 72 hours. Recently, Zhuge et al.^22^ reported that fullerenol, an effective free radical scavenger and antioxidant, could salvage RPE cells from oxidative stress-induced senescence due to its antioxidant effect. They suggested that the protective effect of fullerenol is crucial for the development of new treatment strategies in oxidative stress-associated retinal disorders such as age-related macular degeneration (AMD). In addition, Kernt et al.^23^ investigated the antiapoptotic and cytoprotective effects of idebenone, a benzoquinone derivative that is structurally related to ubiquinone (coenzyme Q10), on optic nerve head astrocytes (ONHA) under oxidative stress. They concluded that idebenone reduced senescence, oxidative stress, and apoptotic cell death in cultured ONHA in vitro. One of the limitations of our study is that we studied the effects of drugs in healthy retinas, not under the drugs’ indicated disease states such as AMD, which would have already had oxidative stress and changes in RPE senescence. Therefore, we do not know the senescence effects of the drugs in already oxidative stress-induced conditions.

Recently, Spitzer et al.^8^ compared the antiproliferative and cytotoxic effects of bevacizumab, pegaptanib, and ranibizumab on different ocular cells. When applied to choroidal epithelial cells, they observed reductions in cell proliferation of 44.1% with ranibizumab versus 38.2% and 35.1% with bevacizumab and pegaptanib, respectively, although the difference between them was not statistically significant. They reported that bevacizumab, pegaptanib, and ranibizumab significantly suppressed choroidal endothelial cell proliferation and concluded that when used at the currently established doses, none of the drugs was superior over the others in respect to endothelial cell growth inhibition. Our study showed no difference in the proliferation rate of the RPE line in the treated groups compared to controls.

Another study compared the effects of ranibizumab, pegaptanib, and bevacizumab at intravitreal dose range on human umbilical vein endothelial cells (HUVEC).^10^ The results indicated that ranibizumab and bevacizumab significantly increased apoptosis of HUVEC, similar to our results in RPE cells. Clinically applied doses of these drugs, but not pegaptanib, caused significantly reduced cellular proliferation without causing cytotoxic effects at all concentrations used. In addition, the active form of VEGF receptor-2 expression was decreased relative to controls after incubation with bevacizumab (to 66% of control values), ranibizumab (78%), and pegaptanib (86%).

Schnichels et al.^11^ investigated the cytotoxicity and antiproliferative activity of aflibercept, bevacizumab, and ranibizumab on different ocular cells (ARPE19, RGC-5, and 661W) and concluded that aflibercept does not cause changes in cell morphology, induce apoptosis, or permanently decrease cell viability, cell density, or proliferation in any cell line or concentration investigated. In addition, aflibercept slightly upregulated or downregulated certain VEGF-related factors, but the changes were not significant when compared to bevacizumab and ranibizumab. The ARPE19 cell line was derived from human RPE in their study, whereas ours was derived from rabbits. VEGF-Trap_R1R2_ (aflibercept) is composed of entirely human sequences and was constructed to bind human VEGF isoforms.^24^ Holash et al.^24^ stated that despite its wholly human nature, VEGF-Trap_R1R2_ binds all species of VEGF tested, from human to chicken VEGF, yet in their study the experiments were shown on mouse, rat, and humans. The conflicting results obtained in our study and that of Schnichels et al.^11^ may be attributable to different responses shown by the cell lines of different origin used in the studies.

It is known that most eyes with AMD require long-term anti-VEGF treatment, and it is the constant neutralization of VEGF, which is crucial for ocular homeostasis, that may lead to adverse effects.^25^

### Study Limitations

One of the limitations of our study is that anti-VEGF drugs are used repeatedly, most commonly administered as 3 monthly loading doses followed by repeated injections monthly or pro re nata (as needed) thereafter. In contrast, our study represents results after a single injection, and longitudinal studies showing the effects of more and repeated injections as in real life should be planned.

## Conclusion

In conclusion, our study reveals that anti-VEGF drugs had no effects on senescence and proliferation of RPE cells. Aflibercept was found to decrease apoptosis and increase cell viability. In contrast, ranibizumab and bevacizumab were observed to increase apoptosis and reduce RPE cell viability. In the literature, there are no studies evaluating the effects of anti-VEGF drugs on senescence. We believe that our study will guide future research in this respect and experimental and preclinical studies will be needed to confirm our in vitro findings.

## References

[1] Aiello LP, Pierce EA, Foley ED, Takagi H, Chen H, Riddle L, Ferrara N, King GL, Smith LE (1995). Suppression of retinal neovascularization in vivo by inhibition of vascular endothelial growth factor (VEGF) using soluble VEGF-receptor chimeric proteins. Proc Natl Acad Sci U S A..

[2] Presta LG, Chen H, O’Connor SJ, Chisholm V, Meng YG, Krummen L, Winkler M, Ferrara N (1997). Humanization of an anti-vascular endothelial growth factor monoclonal antibody for the therapy of solid tumors and other disorders. Cancer Res..

[3] Qaum T, Xu Q, Joussen AM, Clemens MW, Qin W, Miyamoto K, Hassessian H, Wiegand SJ, Rudge J, Yancopoulos GD, Adamis AP (2001). VEGF-initiated blood-retinal barrier breakdown in early diabetes. Invest Ophthalmol Vis Sci..

[4] Gragoudas ES, Adamis AP, Cunningham ET Jr, Feinsod M, Guyer DR;, VEGF Inhibition Study in Ocular Neovascularization Clinical Trial Group (2004). Pegaptanib for neovascular age-related macular degeneration. N Engl J Med..

[5] Wells JA, Glassman AR, Ayala AR, Jampol LM, Bressler NM, Bressler SB, Brucker AJ, Ferris FL, Hampton GR, Jhaveri C, Melia M, Beck RW;, Diabetic Retinopathy Clinical Research Network (2016). Aflibercept, Bevacizumab, or Ranibizumab for Diabetic Macular Edema: Two-Year Results from a Comparative Effectiveness Randomized Clinical Trial. Ophthalmology..

[6] Wang Y, Fei D, Vanderlaan M, Song A (2004). Biological activity of bevacizumab, a humanized anti-VEGF antibody in vitro. Angiogenesis..

[7] Spitzer MS, Wallenfels-Thilo B, Sierra A, Yoeruek E, Peters S, Henke-Fahle S, Bartz-Schmidt KU, Szurman P;, Tuebingen Bevacizumab Study Group (2006). Antiproliferative and cytotoxic properties of bevacizumab on different ocular cells. Br J Ophthalmol..

[8] Spitzer MS, Yoeruek E, Sierra A, Wallenfels-Thilo B, Schraermeyer U, Spitzer B, Bartz-Schmidt KU, Szurman P (2007). Comparative antiproliferative and cytotoxic profile of bevacizumab (Avastin), pegaptanib (Macugen) and ranibizumab (Lucentis) on different ocular cells. Graefes Arch Clin Exp Ophthalmol..

[9] Kaempf S, Johnen S, Salz AK, Weinberger A, Walter P, Thumann G (2008). Effects of bevacizumab (Avastin) on retinal cells in organotypic culture. Invest Ophthalmol Vis Sci..

[10] Carneiro A, Falcao M, Pirraco A, Milheiro-Oliveira P, Falcao-Reis F, Soares R (2009). Comparative effects of bevacizumab, ranibizumab and pegaptanib at intravitreal dose range on endothelial cells. Exp Eye Res..

[11] Schnichels S, Hagemann U, Januschowski K, Hofmann J, Bartz-Schmidt KU, Szurman P, Spitzer MS, Aisenbrey S (2013). Comparative toxicity and proliferation testing of aflibercept, bevacizumab and ranibizumab on different ocular cells. Br J Ophthalmol..

[12] Chang CW, Roque RS, Defoe DM, Caldwell RB (1991). An improved method for isolation and culture of pigment epithelial cells from rat retina. Curr Eye Res..

[13] Kernt M, Neubauer AS, Liegl RG, Hirneiss C, Alge CS, Wolf A, Ulbig MW, Kampik A (2010). Sorafenib prevents human retinal pigment epithelium cells from light-induced overexpression of VEGF, PDGF and PlGF. Br J Ophthalmol..

[14] Dimri GP, Lee X, Basile G, Acosta M, Scott G, Roskelley C, Medrano EE, Linskens M, Rubelj I, Pereira-Smith O, et al (1995). A biomarker that identifies senescent human cells in culture and in aging skin in vivo. Proc Natl Acad Sci U S A..

[15] Avery RL, Bakri SJ, Blumenkranz MS, Brucker AJ, Cunningham ET Jr, D’Amico DJ, Dugel PU, Flynn HW Jr, Freund KB, Haller JA, Jumper JM, Liebmann JM, McCannel CA, Mieler WF, Ta CN, Williams GA (2014). Intravitreal injection technique and monitoring: updated guidelines of an expert panel. Retina..

[16] Cho HJ, Kim HS, Yoo SG, Han JI, Lew YJ, Cho SW, Lee TG, Kim JW (2015). Retinal Pigment Epithelial Tear After Intravitreal Ranibizumab Treatment for Retinal Angiomatous Proliferation. Am J Ophthalmol..

[17] Hata M, Yamashiro K, Oishi A, Ooto S, Tamura H, Miyata M, Ueda-Arakawa N, Kuroda Y, Takahashi A, Tsujikawa A, Yoshimura N (2017). Retinal Pigment Epithelial Atrophy after Anti-Vascular Endothelial Growth Factor Injections for Retinal Angiomatous Proliferation. Retina..

[18] Cam D, Berk AT, Micili SC, Kume T, Ergur BU, Yilmaz O (2017). Histological and Immunohistochemical Retinal Changes Following the Intravitreal Injection of Aflibercept, Bevacizumab and Ranibizumab in Newborn Rabbits. Curr Eye Res..

[19] Malik D, Tarek M, Caceres del Carpio J, Ramirez C, Boyer D, Kenney MC, Kuppermann BD (2014). Safety profiles of anti-VEGF drugs: bevacizumab, ranibizumab, aflibercept and ziv-aflibercept on human retinal pigment epithelium cells in culture. Br J Ophthalmol..

[20] Chen Q, Fischer A, Reagan JD, Yan LJ, Ames BN (1995). Oxidative DNA damage and senescence of human diploid fibroblast cells. Proc Natl Acad Sci U S A..

[21] Serrano M, Lin AW, McCurrach ME, Beach D, Lowe SW (1997). Oncogenic ras provokes premature cell senescence associated with accumulation of p53 and p16INK4a. Cell..

[22] Zhuge CC, Xu JY, Zhang J, Li W, Li P, Li Z, Chen L, Liu X, Shang P, Xu H, Lu Y, Wang F, Lu L, Xu GT (2014). Fullerenol protects retinal pigment epithelial cells from oxidative stress-induced premature senescence via activating SIRT1. Invest Ophthalmol Vis Sci..

[23] Kernt M, Arend N, Buerger A, Mann T, Haritoglou C, Ulbig MW, Kampik A, Hirneiss C (2013). Idebenone prevents human optic nerve head astrocytes from oxidative stress, apoptosis, and senescence by stabilizing BAX/Bcl-2 ratio. J Glaucoma..

[24] Holash J, Davis S, Papadopoulos N, Croll SD, Ho L, Russell M, Boland P, Leidich R, Hylton D, Burova E, Ioffe E, Huang T, Radziejewski C, Bailey K, Fandl JP, Daly T, Wiegand SJ, Yancopoulos GD, Rudge JS (2002). VEGF-Trap: a VEGF blocker with potent antitumor effects. Proc Natl Acad Sci U S A..

[25] Kuroda Y, Yamashiro K, Tsujikawa A, Ooto S, Tamura H, Oishi A, Nakanishi H, Miyake M, Yoshikawa M, Yoshimura N (2016). Retinal Pigment Epithelial Atrophy in Neovascular Age-Related Macular Degeneration After Ranibizumab Treatment. Am J Ophthalmol..

